# Artificial Intelligence in the Radiological Diagnosis of Impacted Maxillary Canines: A Systematic Review

**DOI:** 10.3390/jcm15093373

**Published:** 2026-04-28

**Authors:** Maciej Jedliński, Adam Jedliński, Gabriel Rostkowski, Joanna Janiszewska-Olszowska, Marta Mazur

**Affiliations:** 1 Department of Interdisciplinary Dentistry, Pomeranian Medical University in Szczecin, al. Powstańców Wlkp. 72, 70-111 Szczecin, Poland; joanna.janiszewska.olszowska@pum.edu.pl (J.J.-O.); marta.mazur@uniroma1.it (M.M.); 2 Student Scientific Society at the Department of Interdisciplinary Dentistry, Pomeranian Medical University in Szczecin, al. Powstańców Wlkp. 72, 70-111 Szczecin, Poland; 3 Department of Wellbeing, Health and Environmental Sustainability—BeSSA, Sapienza University of Rome, 02100 Rieti, Italy

**Keywords:** canine, impaction, artificial intelligence, panoramic X-ray, CBCT, efficiency, accuracy

## Abstract

**Objectives:** The aim of this systematic review was to evaluate whether artificial intelligence systems improve the diagnosis and localization assessment of impacted canines in radiological imaging. **Methods:** A systematic literature search was conducted across four electronic databases (MEDLINE/PubMed, Scopus, Embase, and Web of Science) for studies published after 2020, with no language restrictions. Eligible studies were comparative studies involving human subjects that evaluated AI-based systems against experienced clinicians or accepted radiological reference standards for the detection and localization of impacted canines. The risk of bias and applicability were assessed using the adapted QUADAS-3 tool. The review protocol was prospectively registered in PROSPERO (CRD42023487320). **Results:** The search strategy identified 110 records. After the removal of 41 duplicates, 69 articles were screened by title and abstract. Seventeen studies underwent full-text evaluation, and eight studies met the inclusion criteria and were included in the qualitative synthesis. Across the included studies, the overall risk of bias was considered high, primarily due to retrospective study design and limitations in reporting of methodological procedures. **Conclusions:** The available evidence does not provide high-quality studies addressing the studied issue. AI appears to yield more favorable results in CBCT analysis when compared to panoramic radiographs. However, this observation should be interpreted with caution, because the compared studies did not address the same clinical task, since these radiographs were taken in different clinical situations. Further well-designed studies with standardized datasets and external validation are required to better define the potential of artificial intelligence in orthodontic radiological diagnostics.

## 1. Introduction

Canine impaction is defined as the failure of a permanent canine to erupt into its normal functional position within the dental arch during the expected period of development [1]. Impacted maxillary canines represent a significant clinical challenge in orthodontics [2]. Their prevalence is estimated to range between 1 and 3% in the general population and 4–6.5% among individuals seeking orthodontic treatment [3,4,5]. The absence of canine eruption around the age of 10 may be associated with an ectopic position and future impaction, prompting further diagnostic assessment [6].

Radiological imaging plays a central role in the diagnostic workflow. Panoramic radiograph (OPG) is generally considered the first-line radiographic examination for the assessment of suspected canine impaction. Furthermore, OPGs are frequently obtained in early school-age children during the mixed-dentition stage as part of the radiographic assessment of dental development and overall dentition by both general dental practitioners (GPs) and specialists in orthodontics or pediatric dentistry [7]. When radiographic findings confirm or strongly suggest impaction, cone-beam computed tomography (CBCT) may be requested as a second-level imaging modality to provide a more detailed three-dimensional evaluation and support treatment planning [6,8,9].

Impacted canines may be located either palatally or buccally, and several radiographic parameters should be assessed on panoramic radiographs to estimate the likelihood of eruption and guide treatment planning [10]. These parameters typically include:

- The horizontal position of the canine crown relative to the midline and the degree of overlap between the canine crown and the root of the adjacent lateral incisor, which may indicate a risk of canine impaction or lateral incisors’ root resorption, commonly assessed using sector classification from I (the lowest probability of tooth retention) to V (the highest probability of tooth impaction);
- The angulation of the canine long axis relative to the midline;
- The vertical position of the canine cusp tip in relation to the occlusal plane.

Accurate interpretation of these radiographic features requires considerable clinical expertise, particularly because panoramic imaging is affected by image superimposition, geometric distortion, and potential artifacts [11]. In this context, the integration of artificial intelligence (AI) into radiological diagnostics has emerged as a promising approach to support clinicians, especially general practitioners, in image interpretation, potentially improving diagnostic accuracy and reducing human error [12]. AI-assisted systems may also facilitate earlier identification of eruption disturbances, enabling timely referral to orthodontic specialists and the initiation of interceptive treatment when appropriate [13]. If left untreated, ectopic maxillary canines frequently fail to erupt spontaneously and may require complex multidisciplinary management involving surgical exposure and orthodontic traction [14].

Although artificial intelligence has been increasingly investigated in dental imaging and orthodontic diagnostics, the available evidence regarding its role in the detection of impacted canines remains fragmented. To the best of the authors’ knowledge, no systematic review has specifically evaluated the diagnostic performance of artificial intelligence systems in the radiological detection of impacted maxillary canines.

Therefore, the aim of the present study was to systematically review the available literature in order to assess whether the implementation of artificial intelligence systems may improve the diagnosis and localization assessment of impacted maxillary canines in radiological imaging. The null hypothesis is that the introduction of artificial intelligence models for diagnosing impacted canines does not significantly increase diagnostic accuracy and localization assessment.

## 2. Materials and Methods

### 2.1. Search Strategy

The review process was conducted in accordance with the PRISMA statement and the PRISMA 2020 reporting guidelines [15] (Supplementary Material S1), as well as the recommendations of the Cochrane Handbook for Systematic Reviews of Interventions [16]. The literature search was performed on 27 February 2026 in four electronic databases: MEDLINE (via PubMed), Scopus, Embase, and Web of Science. Studies published after 2020 were considered, with no language restrictions applied. The year 2020 was selected as the starting point for capturing studies using modern deep learning approaches and contemporary imaging datasets. Tailored search strategies were developed for each database separately, and the complete search strings are reported in Supplementary Materials S2.

The study protocol was prospectively registered in the PROSPERO database (CRD42023487320). Rayyan software [17,18] was used to facilitate study screening and data extraction.

The research question was structured according to the PICO framework [19] adapted for diagnostic accuracy studies:

- *Population*: Patients undergoing radiological assessment for suspected canine impaction
- *Index test*: Artificial intelligence–based diagnostic systems applied to radiological images
- *Reference standard*: Assessment performed by experienced clinicians and/or accepted radiological reference standards from preselected datasets.
- *Outcome*: Diagnostic accuracy for the detection and localization of impacted canines
- *Study design*: Comparative studies

The primary research question was: What is the performance of artificial intelligence systems in the radiological assessment of impacted maxillary canines across diagnostic and localization assessment applications?

### 2.2. Eligibility Criteria

Studies were considered eligible if they met the following inclusion criteria:

- Comparative studies evaluating artificial intelligence systems for the detection and localization of impacted canines;
- Studies involving human subjects;
- Studies comparing AI-based diagnosis with evaluation performed by experienced clinicians for diagnostic accuracy or accepted radiological reference standards from preselected datasets for localization accuracy;
- Articles published after 2020.

The exclusion criteria were:

- Incomplete studies;
- Case reports;
- Editorials or short communications;
- Narrative or systematic reviews.

### 2.3. Data Extraction

Two reviewers (M.J. and A.J.) screened titles and abstracts independently, using predefined inclusion criteria. Full texts of the selected articles were then assessed for eligibility. Any disagreements were resolved by consultation with a third reviewer (G.R.). A shared spreadsheet was used to compare decisions against the Cochrane Collaboration guidelines [16]. The agreement between the two reviewers was perfect (κ = 1).

### 2.4. Outcome Results

The following data were extracted from each included study: year of publication, study setting, sample size, imaging modality, type of artificial intelligence model, and diagnostic performance metrics, including accuracy, sensitivity (recall), specificity, area under the receiver operating characteristic curve (AUC/ROC), precision, F1-score, and similarity coefficient.

The present synthesis addressed two questions:

(a) Whether AI detects the presence of impacted canines on radiographic imaging;
(b) Whether AI accurately assesses the localization of impacted canines.

### 2.5. Risk of Bias Assessment

The risk of bias and applicability of each study were evaluated using QUADAS-3 [20]. QUADAS-3 comprises four domains (Participants, Index Test, Target Condition, and Analysis). Moreover, this tool provides guidance tailored to review-specific signaling questions. In this review, the signaling questions were adapted to the studies on AI efficiency in radiological image analysis. This approach enabled the authors to address two predefined synthesis questions—diagnostic detection and localization assessment—and QUADAS-3 was applied with review-specific guidance tailored to the type of accuracy estimate reported in each study. Two reviewers independently assessed the included studies using the aforementioned questions. The signaling questions were addressed by employing the response options delineated by the instrument (i.e., yes/probably yes/probably no/no/no information). The signaling questions were adapted to the context of application of AI models to diagnostic studies using radiographic datasets with only binary interpretation possible, which made some of the questions, such as those concerning operator-dependent factors or diagnostic thresholds, not applicable. Signaling questions used in the assessment were as follows:

1. Was a single-gate study design used? (SQ1)
2. Was a consecutive or random sample of participants included? (SQ2)
3. Is the study group a representative sample of the intended-use population? (SQ3)
4. Was the index test conducted and interpreted according to the recommended instructions? (SQ1)
5. Were the index test results interpreted without knowledge of the reference standard results? (SQ2)
6. Were the index test results interpreted with the same information as would be available when the test is used in practice? (SQ3)
7. Does the reference standard adequately identify those with and without the target condition? (SQ1)
8. Was the target condition assessed in all participants? (SQ2)
9. Was the target condition assessed in the same way for all participants? (SQ3)
10. Were all participants included in the analysis? (SQ1)
11. Were missing data handled appropriately? (SQ2)
12. Were the estimates of sensitivity and specificity calculated appropriately? (SQ3)

The four domains are: (1) Participants; (2) Index Test, (3) Target Condition, and (4) Analysis. Domains 1–3 are each judged for risk of bias and applicability; domain 4 is only judged for risk of bias. Each domain was evaluated for its risk of bias, with the following categories employed: low, high, or insufficient information. Where reporting was insufficient to determine compliance with signaling questions, the response was classified as unclear rather than negative. A domain was judged as low risk solely when all relevant signaling questions were answered “yes” or “probably yes”; if not, the final judgment was based on the possible impact of the identified limitations. Disagreements were resolved through discussion with the third reviewer. The QUADAS-3 judgments were tabulated at the study level, with brief rationales provided for each domain. A proper reporting bias assessment was not feasible because of the small number of included studies and the important heterogeneity in employed AI models and the assessed outcomes.

## 3. Results

The search strategy identified 110 records across the four databases. After the removal of 41 duplicates, 69 records were screened by title and abstract. Following the screening process, 52 records were excluded. The remaining 17 articles were assessed for full-text eligibility. All full-text articles were successfully retrieved. Nine studies were excluded after full-text assessment: one because it consisted of short narrative communication (reason 1), one because it was a conference paper lacking methodological details (reason 2), four because they addressed related topics rather than the diagnosis of impacted canines (reason 3), and three studies addressed AI applications in the prediction of maxillary canine impaction, not actual diagnosis (reason 4).

**Figure 1:**
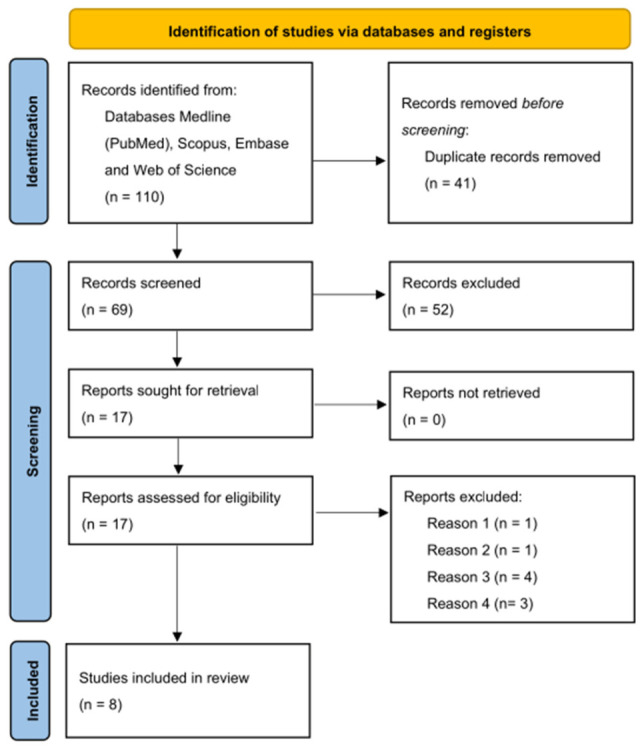
PRISMA 2020 Flow Diagram representing the study selection process.

Finally, eight studies were included in the qualitative synthesis [21,22,23,24,25,26,27,28]. The search results are presented graphically in Figure 1, and the characteristics of the included studies are presented in Table 1.

### 3.1. Results Synthesis

A total of 3441 panoramic radiographs and 914 CBCT scans were analyzed across the included studies. Several studies applied automated preprocessing techniques to panoramic radiographs before AI analysis, whereas others used raw images. In some cases, both processed and unprocessed images derived from the same original dataset were evaluated. The study by Alenezi et al. [22] included both pre- and post-processed images derived from the same original radiographs. Most included studies evaluated AI outputs against experienced clinicians or clinician-derived annotations, whereas selected localization studies used accepted radiological reference standards such as CBCT.

### 3.2. Outcomes

The outcomes reported in the included studies were heterogeneous across different clinical task applications. Diagnostic accuracy studies mainly reported classification metrics such as AUC, sensitivity, specificity, accuracy, precision, recall, and F1-score. Localization assessment studies reported agreement- or measurement-based outcomes, including volumetric variables (voxels), similarity coefficients, and segmentation-related accuracy.

### 3.3. Risk of Bias Assessment

**Figure 2:**
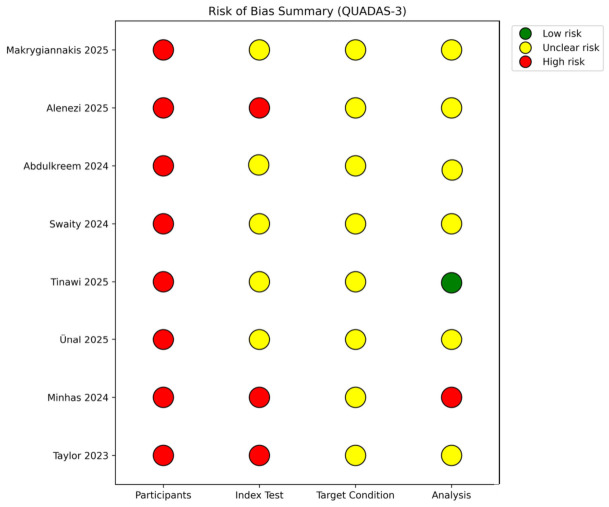
Risk of bias of inluded studies [21,22,23,24,25,26,27,28] in each category.

The exact scoring for each QUADAS-3 domain in each study is presented in Table 2, and the risk of bias assessment is shown in Figure 2. The answers to signaling questions and final risk of bias and applicability concerns are presented in Table 2. The rationale for each judgment in each domain is provided below the table.

#### 3.3.1. Participant Enrollment

Most included studies were retrospective and used selected rather than consecutive clinical datasets. None explicitly demonstrated proper randomized sampling reflective of a routine clinical pathway. Several studies were based on artificially balanced datasets (e.g., equal numbers of impacted versus non-impacted radiographs) or artificially enriched datasets (pre-processing of studied radiographic images). Consequently, the participants’ enrollment was judged to be at high risk of bias throughout the included studies, reflecting potential spectrum bias and limited representativeness.

The applicability concerns were deemed low for all the included studies. The included studies were based on retrospective recruitment of patients of the appropriate age, representing the relevant patient groups.

#### 3.3.2. Index Test

In most studies, the index test consisted of artificial intelligence algorithms evaluated using predefined training and test datasets. For this reason, the risk of bias was assessed as unclear in most of the studies. However, in the case of three studies [22,27,28], the explicit reporting of pre-specified decision thresholds and the calibration process was not clearly described. In these cases, signaling questions were judged as negative. Thus, three studies were judged high risk in this domain [22,27,28]. On the other hand, one study [24], which also used pre-processing of input data, described the cropping and labeling process in detail, so in this case, only an unclear level of risk was identified. The applicability concerns were found to be unclear or high, and this was also due to the possible need to preprocess the input data to make the test work properly.

#### 3.3.3. Target Condition

In most studies, the target condition was defined using appropriate reference standards, including panoramic X-ray or cone beam computed tomography of an appropriate patient group and expert clinician evaluation. When proper adjudication or blinding were not fully described, signaling questions were evaluated as unclear. Overall, this domain was assessed as unclear risk, with none of the studies demonstrating clear evidence of reference standard bias. Applicability concerns were judged low across all the studies, as the reference standards corresponded to accepted definitions of study setting and canine impaction.

#### 3.3.4. Analysis

Most studies used internal validation approaches, such as train-test split or cross-validation, without external validation cohorts, and deemed the risk as unclear. Accordingly, low risk in this domain was assigned only to studies with clearer validation strategies, whereas studies with limited reporting or concerns about optimistic performance estimates were judged to be high risk or insufficiently informative. Consequently, the low risk was attributed to only one study, which also provided validation groups [25]. On the other hand, one study was found to be of high risk [27] due to concerns regarding internal optimization.

## 4. Discussion

Attempts to introduce AI into orthodontic diagnostics represent a significant step toward the automation and objectification of clinical decision-making. The inclusion criteria imposed a restriction on the year of publication to 2020, with the intention of excluding early, inaccurate mathematical models that cannot be compared with the current accuracy and computing power available to modern AI. Although the search strategy allowed inclusion of studies published from 2020 onwards, all eligible studies were published between 2024 and 2025, reflecting the very recent development of AI applications in orthodontic radiological imaging. Each risk-of-bias domain was evaluated as high or unclear in most studies. The principal source of bias arises from retrospective participant selection and non-consecutive sampling. On the other hand, the Target Condition was generally robust, and most Index Test and Analysis concerns stemmed from incomplete reporting rather than methodological flaws. From a clinical perspective, the included studies addressed two closely related yet distinct questions: the detection of impacted canines, primarily on panoramic radiographs, and the localization assessment, primarily in CBCT-based settings.

### 4.1. Impacted Maxillary Canine Diagnosis

OPG remains the primary screening tool in orthodontics due to its low radiation dose and broad imaging range [21,25]. Nevertheless, the interpretation of two-dimensional (2D) images in the context of impacted canines carries a substantial risk of error resulting from the superimposition of anatomical structures and geometric distortion, which represents a major diagnostic limitation. As demonstrated by Abdulkreem et al. [23], raw radiological images often do not allow algorithms to achieve satisfactory results without advanced digital preprocessing. Only through the application of region-of-interest cropping and noise elimination can deep learning models attain high sensitivity [22,23,28]. This indicates that, at the current stage of development, AI applied to panoramic images is not a fully autonomous tool and requires human supervision for the correct calibration of input data.

Another critical issue concerns the timing of diagnosis. Early detection of the risk of tooth impaction (around the age of 10) allows for the implementation of interceptive treatment, which in many cases prevents the need for complex surgical-orthodontic procedures [24]. Unfortunately, in clinical practice, patients with impacted canines are referred to specialists with considerable delay (so-called late referral). This is often due to GPs failing to diagnose the absence of permanent canine eruption within the appropriate developmental window, or misinterpreting 2D images and treating the absence of a tooth as a normal developmental variant [13]. In this regard, AI could serve as an early warning tool, prompting primary care physicians to verify the dentition status after OPG analysis, which is currently still underdeveloped.

### 4.2. Impacted Maxillary Canine Localization Assessment

A significant limitation of AI applications in OPG analysis is the difficulty of unambiguously determining the spatial orientation of an impacted canine, in particular, distinguishing its palatal from its buccal position. As Minhas et al. [27] demonstrated, AI software failed to situate impacted canines in the buccolingual dimension, which is clinically the most relevant parameter. Treatment of palatally and buccally positioned canines differs significantly. Currently, only CBCT provides the clinician with full certainty regarding the tooth’s localization and the state of the surrounding bone [25,27].

It is worth emphasizing the difference between two- and three-dimensional imaging in the context of impacted canine diagnostics. CBCT provides the clinician with complete information through multiplanar reconstruction—precise localization of the canine relative to the roots of adjacent teeth, assessment of the degree of potential resorption, and evaluation of the morphology of the surrounding alveolar bone [24,28]. This also explains the significant heterogeneity within the studies. In primary screening for OPGs, the challenge lies in accurately identifying the presence of impacted canines. Conversely, CBCT provides a different perspective, facilitating the identification of an impacted tooth at first sight. The clinician should therefore focus on determining its precise location in order to provide the most appropriate treatment approach. An experienced specialist, having access to a high-quality CBCT scan, is able to plan treatment independently and with great confidence without AI support—the three-dimensionality of the image eliminates the primary source of diagnostic error, namely the superimposition of structures. The role of AI in CBCT analysis is therefore qualitatively different from its role in OPG: rather than correcting the limitations of the modality, it involves the automation of time-consuming tasks such as tooth and bone segmentation [24,28], which can relieve the clinician of burden but are not a prerequisite for an accurate diagnosis. Despite advances in 2D image analysis, CBCT remains the gold standard in preoperative planning. Studies by Ünal et al. [26] and Swaity et al. [24] confirm that only three-dimensional segmentation allows for the precise determination of the canine’s relationship to the roots of adjacent teeth and assessment of the risk of their resorption.

### 4.3. Clinical Perspective

Beyond the scope of the present synthesis, a particularly promising direction is the application of AI to predict the risk of future occurrence of maxillary canine impaction. However, in this regard, AI also demonstrated more consistent results based on CBCT [29], whereas the prediction models based on OPG require further improvements [30,31]. Thus, an important question arises: in which routine clinical scenario would a pediatric patient undergo CBCT examination? The European and American standards of radiological diagnosis significantly limit the application of CBCT examination in pedodontics, describing it as a last-resort imaging modality [32,33]. For this reason, CBCT should only be performed when justified by the initial examination. Thus, despite the high accuracy reported in CBCT-based AI models, their routine application in everyday clinical practice remains limited due to radiation protection guidelines in pediatric patients.

Notwithstanding the enhancement in the precision of AI systems, the issue of diagnostic responsibility remains fundamental. AI appears most useful as a supportive tool in radiographic detection and in localization-related analysis, rather than as an independent diagnostic system. In ambiguous cases, in the presence of anatomical anomalies or complex anatomical relationships, the final interpretation of the image and the decision regarding treatment must remain the responsibility of the clinician [34]. AI systems based solely on panoramic radiographs currently show variable diagnostic performance and therefore cannot yet replace clinical interpretation by experienced practitioners. On the other hand, AI has the potential to assist medical specialists significantly in workflow-related tasks, such as segmentation of the dentition and in-depth localization analysis [25,26]. The final diagnostic interpretation and treatment planning must remain under the responsibility of experienced clinicians, particularly in cases involving complex anatomical relationships.

### 4.4. Limitations

This study is characterized by several limitations:

1. All included studies were retrospective, which increases the risk of selection bias and limits the strength of the conclusions that can be drawn.
2. There was considerable methodological heterogeneity observed across the included studies, which consists of differences such as AI architectures, imaging modalities, preprocessing procedures, and reported outcome measures, which significantly compromises the possibility to quantitatively synthesize the findings.
3. The included studies analyze the effectiveness of diagnostic methods. Thus, the most appropriate quality assessment tool was QUADAS-3. This approach was considered appropriate because the final review addressed two predefined synthesis questions—diagnostic detection and localization assessment. However, due to the specific characteristics of these studies, an adapted version of this tool had to be implemented.

## 5. Conclusions

The available evidence does not include high-quality studies that demonstrate the effectiveness of artificial intelligence in detecting and localizing impacted canines on radiographic images beyond that of experienced clinicians. AI appears to yield more favorable results in CBCT analysis when compared to panoramic radiographs. However, this observation should be interpreted with caution, because the compared studies did not address the same clinical task, since these radiographs were taken in different clinical situations. Further well-designed studies with standardized datasets and external validation are required to better define the potential of artificial intelligence in orthodontic radiological diagnostics. Consequently, the null hypothesis was not rejected.

## Figures and Tables

**Table 1:** Characteristics of included studies.

Author and Year	Type and Number of Radiological Expositions Analyzed	Comparison Made	Outcome Measured	Result
Makrygiannakis and Kaklamanos, 2025 [21]	200 panoramic X-rays in 4 sets 1. set featured impacted canines, 2. included impacted third molars, 3. contained impacted incisors, premolars, and first and second molars 4. set had no impacted teeth	AI software vs. two expert human observers	Sensitivity—positive predictive value; Specificity—negative predictive value	Cohen’s Kappa coefficient for identifying impacted canines between AI and human experts was 0.397 and 0.914 for third molars. AI-based platform demonstrates potential for identifying impacted third molars, but it remains inadequate for evaluating impacted canines on OPGs.
Alenezi et al. 2025 [22]	182 panoramic X-ray 1. 91 with impacted canines 2. 91 without impacted canines 3. 91 with impacted canines pre-processed 4. 91 without impacted canines pre-processed (same images before and after processing)	8 CNNs (SqueezeNet, GoogLeNet, NASNet-Mobile, ShuffleNet, VGG-16, ResNet 50, DenseNet 201, and Inception V3) vs. two human observers (expert and beginner)	Precision, Sensitivity, Specificity, area under the receiver operating characteristic curve (AUC-ROC)	It is evident that GoogLeNet attained the maximum diagnostic performance. For cropped panoramic radiographs, the values for sensitivity, specificity, accuracy, and AUC-ROC were 0.91, 0.97, 0.94, and 0.99, respectively. For uncropped images, the corresponding values were 0.81, 0.85, 0.83, and 0.90. Repeated training of GoogLeNet yielded values of sensitivity 1.00, specificity 0.91, accuracy 0.95, and AUC-ROC 0.98 for cropped images, compared with 0.85, 0.85, 0.85, and 0.93 for uncropped images. The findings indicate that automated preprocessing substantially improves classification accuracy, maintaining correct discrimination.
Abdulkreem et al. 2024 [23]	1490 panoramic radiograph in 3 sets 1. 108 with impacted canines 2. 108 without impacted canines 3. 91 with impacted canines pre-processed 4. 91 without impacted canines pre-processed 5. 1092 as training dataset	AI software vs. two expert human observers	Precision, Recall, F1-score, mean Average Precision, AUC	In the absence of preprocessing, the CNN (SqueezeNet) attained an AUC of 0.84 for the classification of impacted versus non-impacted maxillary canines on panoramic radiographs. The utilization of specific landmarks to facilitate automated radiograph cropping led to a reduction in cropping, resulting in an augmented AUC value of 0.96. The findings of this study suggest that the implementation of automated preprocessing significantly enhances the efficacy of AI in identifying impacted canines, in comparison with the utilization of unprocessed inputs.
Swaity et al. 2024 [24]	316 CBCT scans: 1. 50 scans as a training dataset 2. 50 scans as a test dataset	AI software vs. two expert human observers	Accuracy, in voxel superimposition correctness	The CNN-based automated segmentation demonstrated almost perfect agreement with semi-automated segmentations, reaching a Dice similarity coefficient of 0.99 ± 0.02 and an intersection over union of 0.99 ± 0.04. The mean 95% Hausdorff distance was 0.04 ± 0.08 mm, with a root mean square surface difference of 0.05 ± 0.25 mm. The intra- and inter-observer repeatability of refinement was found to be high (ICC = 0.992 and 0.986). The automated approach exhibited a mean processing time of 21 s, in comparison to the 582 s required by semi-automated segmentation. This indicates that AI was approximately 24 times faster while maintaining a level of accuracy comparable to that of experts.
Tinawi et al. 2025 [25]	316 CBCT scans: 1. 221 scans as training dataset 2. 95 scans as test dataset	AI software vs. two expert human observers	Canine position, distance to occlusal plane, sagittal overlap, roll, pitch, severity index, and root damage to adjacent teeth	The AI-based system demonstrated a high level of accuracy in comparison with manual clinician measurements, achieving a mean detection success rate of 96.2%. The identification of landmarks revealed a range of success rates, from 92.3% (left canine apex) to 100% (right canine apex), with an error threshold of 4 mm. The mean error values were 0.40–1.34 mm. The AI-based diagnostic system was validated to accurately identify impacted canine position, severity, and overlap with adjacent teeth.
Ünal et al. [26]	159 CBCT scans: 1. 145 scans as training dataset 2. 14 scans as test dataset	Difference between voxels marked by software and individualized by two expert human observers in the training dataset	Accuracy, Sensitivity, Specificity in voxel superimposition correctness	The recall value of the model was found to be 0.90. The model’s precision value was found to be lower than its recall, calculated at 0.82. The automatic segmentation accuracy was found to be 0.84.
Minhas et al. 2024 [27]	123 panoramic X-rays and 123 CBCTs: 1. 74 patients with impacted canines 2. 49 patients without impacted canines	3D reconstruction of panoramic X-ray vs. actual position of impacted canine on CBCT (reference standard)	Structure similarity index in the form of buccolingual and mesiodistal accuracy	The mean SSIM was 0.71 (0.63–0.8), with only 41% accuracy in the buccolingual dimension and 55% in the mesiodistal dimension. Although the software produced diagnostically useful visual output, the CBCT was still required for accurate positioning.
Taylor 2023 [28]	1446 panoramic X-ray in pre-processed 2 sets: 1. 723 patients with impacted canines 2. 723 patients without impacted canines	AI software with 2 convolutional layers of DenseNet-121 and VGG-19 tested separately vs. two expert human observers	Accuracy	Peak accuracy ranged from 62% for the DenseNet-12117 algorithm with 40 prototypes to 92% on the DenseNet-121 with 200 prototypes.

**Table 2:** Risk of bias assessment according to the QUADAS-3 tool.

Study (Author, Year)	Participants SQ1	Participants SQ2	Participants SQ3	Participants RoB L/H/II	Participants App L/H/II	Index Test SQ1	Index Test SQ2	Index Test SQ3	Index Test RoB L/H/II	Index Test App L/H/II
Makrygiannakis and Kaklamanos, 2025 [21]	N	Y	II	H	L	Y	II	Y	II	II
Alenezi et al., 2025 [22]	N	N	II	H	L	Y	PN	PN	H	H
Abdulkreem et al., 2024 [23]	N	N	II	H	L	Y	PN	Y	II	H
Swaity et al., 2024 [24]	N	Y	II	H	L	Y	II	Y	II	II
Tinawi et al., 2025 [25]	II	N	II	H	L	Y	II	Y	II	II
Ünal et al., 2025 [26]	N	N	II	H	L	Y	II	Y	II	II
Minhas et al., 2024 [27]	N	N	N	H	L	Y	PN	PN	H	H
Taylor 2023 [28]	N	N	II	H	L	Y	PN	PN	H	H
**Study (Author, Year)**	**Target Condition** **SQ1**	**Target Condition** **SQ2**	**Target Condition** **SQ3**	**Target Condition** **RoB** **L/H/II**	**Target Condition** **App** **L/H/II**	**Analysis** **SQ1**	**Analysis** **SQ2**	**Analysis** **SQ3**	**Analysis** **RoB** **L/H/II**	
Makrygiannakis and Kaklamanos, 2025 [21]	Y	II	II	II	L	Y	Y	II	II	
Alenezi et al., 2025 [22]	Y	II	II	II	L	Y	Y	II	II	
Abdulkreem et al., 2024 [23]	Y	II	II	II	L	Y	H	Y	Il	
Swaity et al., 2024 [24]	Y	Y	II	II	L	Y	Y	Y	L	
Tinawi et al., 2025 [25]	Y	Y	II	II	L	Y	Y	II	II	
Ünal et al., 2025 [26]	Y	Y	II	II	L	Y	Y	II	II	
Minhas et al., 2024 [27]	Y	II	II	II	L	Y	N	N	H	
Taylor 2023 [28]	Y	II	II	II	L	Y	Y	II	II	

Key: SQ = signaling question, RoB—Risk of Bias; App—applicability; Y = yes, PN = probably no, N = no, II = unclear/insufficient information, L = low, H = high.

## Data Availability

All data are available from the corresponding author upon reasonable request.

## Supplementary Materials

The following supporting information can be downloaded at: https://www.mdpi.com/article/10.3390/jcm15093373/s1, Supplementary Materials S1: PRISMA 2020 checklists; Supplementary Material S2: Detailed information regarding the search process.

## Abbreviations

The following abbreviations are used in this manuscript:

GP	General practitioner
OPG	Orthopantomogram
CBCT	Cone-beam computed tomography
AI	Artificial intelligence
