# Supplementary material for: Artificial Intelligence in the Radiological Diagnosis of Impacted Maxillary Canines: A Systematic Review
Source: J Clin Med. 2026 Apr 28;15(9):3373. doi: 10.3390/jcm15093373 (PMC13164204; doi:10.3390/jcm15093373)
Supplement: Supplementary file 1 [file jcm-15-03373-s001.zip › Supplementary Material S2.pdf]

Supplementary Material S2.

A) Exact search string in each database

PubMed - ("Cuspid"[MeSH] OR impacted canine*[Title/Abstract] OR impacted maxillary canine*[Title/Abstract] OR unerupted canine*[Title/Abstract]) AND ("Radiography"[MeSH] OR "Diagnostic Imaging"[MeSH] OR "Cone-Beam Computed Tomography"[MeSH] OR radiolog*[Title/Abstract] OR imaging[Title/Abstract] OR radiograph*[Title/Abstract] OR CBCT[Title/Abstract] OR panoramic[Title/Abstract] OR cephalometric[Title/Abstract]) AND ("Artificial Intelligence"[MeSH] OR "Machine Learning"[MeSH] OR "Deep Learning"[MeSH] OR artificial intelligence[Title/Abstract] OR machine learning[Title/Abstract] OR deep learning[Title/Abstract] OR neural network*[Title/Abstract] OR algorithm*[Title/Abstract]) AND (diagnosis[Title/Abstract] OR diagnostic*[Title/Abstract] OR detection[Title/Abstract] OR assessment[Title/Abstract] OR localization[Title/Abstract] OR location[Title/Abstract])

Scopus- TITLE-ABS-KEY ((impacted AND canine*) OR (unerupted AND canine*)) AND TITLE-ABS-KEY (radiolog* OR imaging OR radiograph* OR cbct OR panoramic OR cephalometric) AND TITLE-ABS-KEY ("artificial intelligence" OR "machine learning" OR "deep learning" OR "neural network*" OR algorithm*) AND TITLE-ABS-KEY (diagnosis OR diagnostic* OR detection OR assessment OR localization OR location)

Embase - (('cuspid'/exp OR impacted) AND canine*:ti,ab,kw OR unerupted) AND canine*:ti,ab,kw AND ('diagnostic imaging'/exp OR 'radiography'/exp OR 'cone beam computed tomography'/exp OR radiolog*:ti,ab,kw OR imaging:ti,ab,kw OR radiograph*:ti,ab,kw OR panoramic:ti,ab,kw OR cephalometric:ti,ab,kw) AND (((('artificial intelligence'/exp OR 'machine learning'/exp OR 'deep learning'/exp OR artificial) AND intelligence:ti,ab,kw OR machine) AND learning:ti,ab,kw OR deep) AND learning:ti,ab,kw OR 'neural network*':ti,ab,kw OR algorithm*:ti,ab,kw) AND (diagnosis:ti,ab,kw OR diagnostic*:ti,ab,kw OR detection:ti,ab,kw OR assessment:ti,ab,kw OR localization:ti,ab,kw OR location:ti,ab,kw)

Web of Science- ALL=((impacted AND canine*) OR (unerupted AND canine*))

AND ALL=(radiolog* OR imaging OR radiograph* OR CBCT OR panoramic OR cephalometric) AND ALL=("artificial intelligence" OR "machine learning" OR "deep learning" OR "neural network*" OR algorithm*) AND ALL=(diagnosis OR diagnostic* OR detection OR assessment OR localization OR location) .

B) Tables of excluded articles

Studies after title and abstract analysis

Title	Author	Journal	Reason for exclusion
Deep Learning in Diagnosis of Dental Anomalies and Diseases: A Systematic Review	Sivari, E. and Senirkentli, G.B. and Bostanci, E. and Güzel, M.S. and Acici, K. and Aşuroğlu, T.	Diagnostics	Study type - review
Comparison of error rates between four pretrained DenseNet convolutional neural network models and 13 board-certified veterinary radiologists when evaluating 15 labels of canine thoracic radiographs	Hespel, H. and Emilie, B. and Alois, D.L.C. and Michelle, A. and Kate, A. and Mylene, A. and David, B. and Marie, D.S. and Jason, F. and Eric, G. and Séamus, H. and Kevin, K. and Alison, L. and Megan, M. and Hester, M. and Jaime, R.J. and Zhu, X. and Micaela, Z. and Federica, M.	Veterinary Radiology and Ultrasound	Out of scope - animal study
Machine (Deep) learning for characterization of craniofacial variations	Chen, S. and Wu, T.-J. and Wu, T.-H. and Pastewait, M. and Zheng, A. and Wang, L. and Wang, X. and Ko, C.-C.	Machine Learning in Dentistry - Book chapter	Out of scope - not impacted canine diagnosis
The use of cone beam CT(CBCT) in differentiation of true from mimicking Eagle's syndrome	Tijanić, M. and Burić, N. and Burić, K.	International Journal of Environmental Research and Public Health	Out of scope - not impacted canine diagnosis
Determinants of maxillary canine impaction: Retrospective clinical and radiographic study	Laurenziello, M. and Montaruli, G. and Gallo, C. and Tepedino, M. and Guida, L. and Perillo, L. and Troiano, G. and Lo Muzio, L.L. and Ciavarella, D.	Journal of Clinical and Experimental Dentistry	Out of scope - not referring to AI implementation
Novel Time Resolved Optical and Machine Learning Methods for Label Free Biomedical Imaging	Mugdha, Arya Chowdhury Z.	Book chapter	Out of scope - not impacted canine diagnosis
Cross-Domain Generalization of Deep Learning-Based Image	Wilm, Frauke Eva	Book chapter	Out of scope - not impacted canine diagnosis

Analysis Algorithms in Histopathology			
Deep learning for necrosis detection using canine perivascular wall tumour whole slide images	Rai, Taranpreet and Morisi, Ambra and Bacci, Barbara and Bacon, Nicholas J. and Dark, Michael J. and Aboellail, Tawfik and Thomas, Spencer Angus and Bober, Miroslaw and La Ragione, Roberto and Wells, Kevin	SCIENTIFIC REPORTS	Out of scope - not impacted canine diagnosis
AI-driven canine cataract detection: a machine learning approach using support vector machine	Jones, Aida and Vijayan, Thulasi Bai	JOURNAL OF THE CHINESE INSTITUTE OF ENGINEERS	Out of scope - animal study
Classification of the quality of canine and feline ventrodorsal and dorsoventral thoracic radiographs through machine learning	Tahghighi, Peyman and Appleby, Ryan B. and Norena, Nicole and Ukwatta, Eran and Komeili, Amin	VETERINARY RADIOLOGY & ULTRASOUND	Out of scope - animal study
Automated Detection of Canine Babesia Parasite in Blood Smear Images Using Deep Learning and Contrastive Learning Techniques	Baruah, Dilip Kumar and Boruah, Kuntala and Barman, Nagendra Nath and Deka, Abhijit and Bharali, Arpita and Buragohain, Lukumoni	PARASITOLOGIA	Out of scope - animal study
Exploring deep learning strategies for intervertebral disc herniation detection on veterinary MRI	Huang, Shoujin and Deng, Guoxiong and Kang, Yan and Li, Jianzhong and Li, Jingyu and Lyu, Mengye	SCIENTIFIC REPORTS	Out of scope - animal study
Contemporary CBCT diagnostics-discovery of a new artery with possible impact on surgical planning: the anterior superior palatal alveolar artery	Kurrek, Andreas and Troedhan, Angelo and Konschake, Marko	SURGICAL AND RADIOLOGIC ANATOMY	Out of scope - not impacted canine diagnosis
Improved Imaging of Fibrosis in Atrial Fibrillation	DIBELLA, EDWARD VR; Shireen Youssef Elhabian; Eugene Kholmovski; Ravi Ranjan		Out of scope - not impacted canine diagnosis
A combined machine-learning and graph-based framework for the 3-D automated segmentation of	Antony, Bhavna Josephine Z.		Out of scope - not impacted canine diagnosis

retinal structures in SD-OCT images			
Deep learning algorithms out-perform veterinary pathologists in detecting the mitotically most active tumor region	Aubreville, Marc and Bertram, Christof A. and Marzahl, Christian and Gurtner, Corinne and Dettwiler, Martina and Schmidt, Anja and Bartenschlager, Florian and Merz, Sophie and Fragoso, Marco and Kershaw, Olivia and Klopfleisch, Robert and Maier, Andreas	SCIENTIFIC REPORTS	Out of scope - animal study
Electromechanical Wave Imaging in the Clinic: Localization of Atrial and Ventricular Arrhythmias and Quantification of Cardiac Resynchronization Therapy Response	Melki, Lea Z.		Out of scope - not impacted canine diagnosis
A combined machine-learning and graph-based framework for the segmentation of retinal surfaces in SD-OCT volumes	Antony, Bhavna J. and Abramoff, Michael D. and Harper, Matthew M. and Jeong, Woojin and Sohn, Elliott H. and Kwon, Young H. and Kardon, Randy and Garvin, Mona K.	BIOMEDICAL OPTICS EXPRESS	Out of scope - not impacted canine diagnosis
PHOTOACOUSTIC ULTRASOUND IMAGING TO DISTINGUISH BENIGN FROM MALIGNANT PROSTATE CANCER	SANO, MICHAEL		Out of scope - not impacted canine diagnosis
Automated Computational Detection, Quantitation, and Mapping of Mitosis in Whole-Slide Images for Clinically Actionable Surgical Pathology Decision Support.	Puri, Munish and Hoover, Shelley B and Hewitt, Stephen M and Wei, Bih-Rong and Adissu, Hibret Amare and Halsey, Charles H C and Beck, Jessica and Bradley, Charles and Cramer, Sarah D and Durham, Amy C and Esplin, D Glen and Frank, Chad and Lyle, L	Journal of pathology informatics	Out of scope - not impacted canine diagnosis

	Tiffany and McGill, Lawrence D and Sanchez, Melissa D and Schaffer, Paula A and Traslavina, Ryan P and Buza, Elizabeth and Yang, Howard H and Lee, Maxwell P and Dwyer, Jennifer E and Simpson, R Mark		
SBIR Phase I: Point-of-Care Periodontal Imaging	Khazaeinezhad, Reza		Out of scope - not impacted canine diagnosis
Investigating the potential of untrained convolutional layers and pruning in computational pathology	Rai, T. and Papanikolaou, I. and Dave, N. and Morisi, A. and Bacci, B. and Thomas, S. A. and La Ragione, R. and Wells, K.	MEDICAL IMAGING 2023	Out of scope - not impacted canine diagnosis
Ultrasound elastographic imaging of thermal lesions and temperature profiles during radiofrequency ablation	Techavipoo, Udomchai Z2 -		Out of scope - not impacted canine diagnosis
DEVELOPMENT AND EVALUATION OF TRACKING ALGORITHMS FOR CARDIAC WALL-MOTION ANALYSIS USING PHASE-VELOCITY MR-IMAGING	CONSTABLE, RT and RATH, KM and SINUSAS, AJ and GORE, JC	MAGNETIC RESONANCE IN MEDICINE	Out of scope - not impacted canine diagnosis
SUBHARMONIC IMAGING AND PRESSURE ESTIMATION FOR MONITORING NEOADJUVANT CHEMOTHERAPY	FORSBERG, FLEMMING		Out of scope - not impacted canine diagnosis
Large-Scale Comparative Analysis of Canine and Human Osteosarcomas Uncovers Conserved Clinically Relevant Tumor Microenvironment Subtypes	Patkar, Sushant and Mannheimer, Joshua and Harmon, Stephanie A. and Ramirez, Christina J. and Mazcko, Christina N. and Choyke, Peter L. and Brown, Gregory Thomas	CLINICAL CANCER RESEARCH	Out of scope - not impacted canine diagnosis

	and Turkbey, Baris and Leblanc, Amy K. and Beck, Jessica A.		
Delayed tooth eruption: Pathogenesis, diagnosis and treatment. A literature review	Suri, L and Gagari, E and Vastardis, H	AMERICAN JOURNAL OF ORTHODONTICS AND DENTOFACIAL ORTHOPEDICS	Study type - review
Accessing Developmental Information of Fossil Hominin Teeth Using New Synchrotron Microtomography-Based Visualization Techniques of Dental Surfaces and Interfaces	Le Cabec, Adeline and Tang, Nancy and Tafforeau, Paul	PLOS ONE	Out of scope - not impacted canine diagnosis
Emotion Classification in Domestic Dogs Using Computer Vision Based on the Dog's Body and Face	Besa, Jianina Vennice T. and Ignacio, Jericho Ivan D. and Zaguirre, Stephanie Anne A. and De Goma, Joel C. and Villaluz, Alberto C. and Assoc Computing Machinery	PROCEEDINGS OF THE 2024 9TH INTERNATIONAL CONFERENCE ON INTELLIGENT INFORMATION TECHNOLOGY, ICIIT 2024	Out of scope - animal study
Quantitative modeling of the accuracy in registering preoperative patient-specific anatomic models into left atrial cardiac ablation procedures	Rettmann, Maryam E. and Holmes, David R., III and Kwartowitz, David M. and Gunawan, Mia and Johnson, Susan B. and Camp, Jon J. and Cameron, Bruce M. and Dalegrave, Charles and Kolasa, Mark W. and Packer, Douglas L. and Robb, Richard A.	MEDICAL PHYSICS	Out of scope - not impacted canine diagnosis
Acetabular Coverage Area Occupied by the Femoral Head as an Indicator of Hip Congruency	Franco-Goncalo, Pedro and da Silva, Diogo Moreira and Leite, Pedro and Alves-Pimenta, Sofia and Colaco, Bruno and Ferreira, Manuel and Goncalves, Lio and Filipe, Vitor and McEvoy, Fintan and Ginja, Mario	ANIMALS	Out of scope - not impacted canine diagnosis
Feasibility study of portable multi-energy computed tomography with photon-	Lee, Chang-Lae and Hong, Key Jo and Kim, Namwoo and Han, Kwanhee and	SCIENTIFIC REPORTS	

counting detector for preclinical and clinical applications	Kim, Dongkyu and Jung, Hoe-Su and Lee, Sangmin and Park, Junyoung and Lee, Kyoung-Yong and Lee, Jee Eun and Choi, Yuna and Cho, Minkook		
MRI-Guided Target Motion Assessment using Dynamic Automatic Segmentation	Saenz, Daniel L. Z2 -		Out of scope - not impacted canine diagnosis
Table_1_Evaluation of Sella Turcica Bridging and Morphology in Different Types of Cleft Patients.xlsx	Alam, Mohammad Khursheed and Alfawzan, Ahmed Ali	Figshare	Out of scope - not impacted canine diagnosis
Abnormal Left Ventricular Mechanics of Ventricular Ectopic Beats Insights Into Origin and Coupling Interval in Premature Ventricular Contraction-Induced Cardiomyopathy	Potfay, Jonathan and Kaszala, Karoly and Tan, Alex Y. and Sima, Adam P. and Gorcsan, John, III and Ellenbogen, Kenneth A. and Huizar, Jose F.	CIRCULATION-ARRHYTHMIA AND ELECTROPHYSIOLOGY	Out of scope - not impacted canine diagnosis
Large Scale Comparative Deconvolution Analysis of the Canine and Human Osteosarcoma Tumor Microenvironment Uncovers Conserved Clinically Relevant Subtypes.	Patkar, Sushant and Mannheimer, Josh and Harmon, Stephanie and Mazcko, Christina and Choyke, Peter and Brown, G Tom and Turkbey, Baris and LeBlanc, Amy and Beck, Jessica	bioRxiv : the preprint server for biology	Out of scope - not impacted canine diagnosis
A Retrospective Study of Clinical and Histopathological Features of 81 Cases of Canine Apocrine Gland Adenocarcinoma of the Anal Sac: Independent Clinical and Histopathological Risk Factors Associated with Outcome	Wong, Hannah and Byrne, Stephanie and Rasotto, Roberta and Drees, Randi and Taylor, Angela and Priestnall, Simon L. and Leo, Chiara	ANIMALS	Out of scope - animal study
III: Small: Visualizing Robust Features in Vector and Tensor Fields	Phillips, Bei		Out of scope - not impacted canine diagnosis
Classification and numbering of teeth in multi-slice CT images	Hosntalab M and Aghaeizadeh Zoroofi R and Abbaspour Tehrani-Fard A and Shirani G	International journal of computer assisted radiology and surgery	

using wavelet-Fourier descriptor.			
Diagnostic algorithm for localization and complexity assessment of impacted maxillary canines	Aliyeva, G. and Seyfullayeva, S. and Musayeva, A.	J. World Fed. Orthod.	Out of scope - not referring to AI implementation
A fully automated deep learning framework for age estimation in adults using periapical radiographs of canine teeth	Upalananda, W. and Phisutphithayakun, C. and Assawasuksant, P. and Tanwattana, P. and Prasatkaew, P.	Int J Legal Med	Out of scope - not impacted canine diagnosis
Center of resistance of maxillary canines: a 3D computational model for orthodontic applications	Gandhi, V. and Abu Arqub, S. and Pierce, D. and Yadav, S. and Upadhyay, M.	Eur J Orthod	Out of scope - not impacted canine diagnosis
Applications of artificial intelligence in diagnosis and treatment planning of orthodontics: a narrative review	Azizi, S. and Hatampoor, S. and Tahamtan, S.	Saudi Dent. J.	Study type - review
A Cone-Beam Computed Tomography-Based Assessment of Safe Zones for Orthodontic Mini-Implant Placement in the Lateral Maxilla: A Retrospective Morphometric Study	Jakovljevic, I. and Milanovic, P. and Vasiljevic, M. and Milanovic, J. and Stevanovic, M.Z. and Jovicic, N. and Stepovic, M. and Ristic, V. and Selakovic, D. and Rosic, G. and Arnaut, A.	Diagnostics	Out of scope - not impacted canine diagnosis
Image Processing for Tooth Type Classification using Deep Learning	Çelik, B. and Ulus, F. and Savaştaer, E.F. and Genç, M.Z. and Çelik, M.E.	Derin Öğrenme Kullanılarak Diş Tipi Sınıflandırması için Görüntü İşleme	Out of scope - not impacted canine diagnosis
How successful is the CatBoost classifier in diagnosing different dental anomalies in patients via sella turcica and vertebral morphologic alteration?	Gonca, M. and Gul, B.B. and Sert, M.F.	BMC Med Inform Decis Mak	Out of scope - not impacted canine diagnosis
Assessing the Effectiveness of Artificial Intelligence Models for Detecting Alveolar Bone Loss in Periodontal Disease: A Panoramic Radiograph Study	Uzun Saylan, B.C. and Baydar, O. and Yeşilova, E. and Kurt Bayrakdar, S. and Bilgir, E. and Bayrakdar, İ.Ş. and Çelik, Ö. and Orhan, K.	Diagn.	Out of scope - not impacted canine diagnosis

Tooth recognition of 32 tooth types by branched single shot multibox detector and integration processing in panoramic radiographs	Morishita, T. and Muramatsu, C. and Seino, Y. and Takahashi, R. and Hayashi, T. and Nishiyama, W. and Zhou, X. and Hara, T. and Katsumata, A. and Fujita, H.	J. Med. Imaging	Out of scope - not impacted canine diagnosis
Estimating the size of unerupted teeth: Moyers vs deep learning	Camcı, H. and Salmanpour, F.	Am J Orthod Dentofacial Orthop	Out of scope - not impacted canine diagnosis
Tooth detection and classification on panoramic radiographs for automatic dental chart filing: improved classification by multi-sized input data	Muramatsu, C. and Morishita, T. and Takahashi, R. and Hayashi, T. and Nishiyama, W. and Ariji, Y. and Zhou, X. and Hara, T. and Katsumata, A. and Ariji, E. and Fujita, H.	Oral Radiol.	Out of scope - not impacted canine diagnosis
Tooth numbering on dental panoramic radiographs using multiclass detection network	Muramatsu, C. and Morishita, T. and Katsumata, A. and Takahashi, R. and Hayashi, T. and Nishiyama, W. and Ariji, Y. and Zhou, X. and Hara, T. and Ariji, E. and Fujita, H.	Int. J. Comput. Assisted Radiol. Surg.	Out of scope - not impacted canine diagnosis
TACTICS OF THE TREATMENT OF TEETH TRANSPOSITION (CASE REPORTS)	Flis, P. and Filonenko, V. and Doroshenko, N.	Georgian Med News	Out of scope - not impacted canine diagnosis
ANet: A Transformer-based Edge Representation Learning Network for Canine X-ray Verification	Lee, In-Gyu, Oh, Jun-Young, Choi, Hyewon, Kam, Tae-Eui, Lee, Namsoon, Hyun, Sang-Hwan, Lee, Euijong, Jeong, Ji-Hoon	MEDICAL IMAGE COMPUTING AND COMPUTER ASSISTED INTERVENTION	Out of scope - animal study

Studies excluded after full-text analysis

**Title**	**Author**	**Journal**	**Reason for exclusion**
Chat Generative Pretrained Transformer-4.0 in determining labiolingual	Kleebayoon, A. and Wiwanitkit, V.	American Journal of Orthodontics and Dentofacial Orthopedics	Editorial, short communication

localization of maxillary impacted canines and presence of resorption in incisors through panoramic radiographs: A correspondence			
Detection and classification of dental pathologies using faster-RCNN in orthopantomogram radiography image	Laishram, A. and Thongam, K.	Conference paper	Editorial, short communication
Deep learning driven segmentation of maxillary impacted canine on cone beam computed tomography images	Swaity, Abdullah and Elgarba, Bahaaeldeen M. and Morgan, Nermin and Ali, Saleem and Shujaat, Sohaib and Borsci, Elena and Chilvarquer, Israel and Jacobs, Reinhilde	SCIENTIFIC REPORTS	Do not directly correspond to the diagnosis of impacted canines
Impact of Image Context on Deep Learning for Classification of Teeth on Radiographs	Krois, Joachim and Schneider, Lisa and Schwendicke, Falk	JOURNAL OF CLINICAL MEDICINE	Do not directly correspond to the diagnosis of impacted canines
Factors affecting the clinical approach to impacted maxillary canines: A Bayesian network analysis.	Nieri M and Crescini A and Rotundo R and Baccetti T and Cortellini P and Pini Prato GP	American Journal of Orthodontics and Dentofacial Orthopedics	Do not directly correspond to the diagnosis of impacted canines
A hierarchical deep learning approach for diagnosing impacted canine-induced root resorption via cone-beam computed tomography	Pirayesh, Z. and Mohammad-Rahimi, H. and Motamedian, S.R. and Amini Afshar, S. and Abbasi, R. and Rohban, M.H. and Mahdian, M. and Ghazizadeh Ahsaie, M. and Iranparvar Alamdari, M.	BMC Oral Health	Do not directly correspond to the diagnosis of impacted canines
Predicting the Risk of Maxillary Canine Impaction Based on Maxillary Measurements Using Supervised Machine Learning	De Araujo, C.M.; Freitas, P.F.D.J.; Ferraz, A.X.; et al	Orthod. Craniofac. Res.	AI applications in prediction of maxillary canine impaction
Deep learning-assisted comparison of different models for predicting maxillary canine	Zhang, C.; Zhu, H.; Long, H.; Shi, Y.; Guo, J.; You, M	Am. J. Orthod. Dentofacial Orthop.	AI applications in prediction of maxillary canine impaction

impaction on panoramic radiography			
Deep learning-based prediction of buccal, middle and palatal orientations of impacted maxillary canines using panoramic radiographs: A pilot study	Cokakoglu, S.; Tosun, S.; Ozic, M.U.	BMC Oral Health	AI applications in prediction of maxillary canine impaction
